# Multilevel analysis of early initiation of breastfeeding in Ethiopia

**DOI:** 10.3389/fpubh.2024.1393496

**Published:** 2024-05-15

**Authors:** Nuru Mohammed Hussen, Tigabu Hailu Kassa, Getnet Mamo Habtie

**Affiliations:** Department of Statistics, Samara University, Semera, Ethiopia

**Keywords:** early initiation of breastfeeding, Ethiopia, demographic survey, regression analysis, public health

## Abstract

**Introduction:**

Breast milk is the ideal food for the infant and is associated with various public health benefits for both the infant and the mother. The recommended time for early initiation of breastfeeding is within one hour after birth. The prevalence of early initiation of breastfeeding was lower than the plan of the Ethiopian Ministry of Health Sector Development program. Thus, the main objective of this study was to identify individual and group-level factors associated with the early initiation of breastfeeding in Ethiopia.

**Methods:**

Secondary data on children was obtained from the 2019 Ethiopia mini-demographic and health survey. The survey was a population-based cross-sectional study and was downloaded from the Measure Demographic and Health Survey website (http://www.measuredhs.com). The study included a random sample of 2,125 last-born infants who were born within 24 months before the survey. A multilevel binary logistic regression analysis was employed to identify the factors associated with the early initiation of breastfeeding in Ethiopia. Statistical data was analyzed using the Statistical Analysis System (SAS 9.4).

**Results:**

The prevalence of early breastfeeding initiation was 72%. The higher preceding birth interval (AOR = 1.18, 95% CI: 1.1076, 1.5451), the higher gestational age of infants (AOR = 1.38, 95% CI: 1.2796, 1.4782), the higher number of antenatal care visits (AOR = 1.26, 95% CI: 1.2340, 1.2934), delivery at a health facility (AOR = 1.60, 95% CI: 1.4585, 1.7515), vaginal delivery (AOR = 1.11, 95% CI: 1.1019, 1.1123), mothers with primary education (AOR = 1.14, 95% CI: 1.0204, 1.2738), mothers with secondary education (AOR = 1.54, 95% CI: 1.4678, 1.6190), and mothers with higher education (AOR = 2.62, 95% CI: 2.2574, 3.0526) were associated with higher odds of early initiation of breastfeeding. Being a rural dweller (AOR = 0.63, 95% CI: 0.5684, 0.7038) and the age of mothers (AOR = 0.44, 95% CI: 0.3921, 0.4894) were associated with lower odds of early initiation of breastfeeding.

**Conclusion:**

Since the prevalence of early initiation of breastfeeding was minimal among rural mothers who delivered their child by caesarean section, this study strongly suggests special supportive care for these mothers.

## Introduction

Breast milk is the ideal food for the infant and is associated with lower neonatal mortality, prevents morbidities such as diarrhea, pneumonia, and neonatal sepsis, and may reduce obesity and diabetes later in life (1, 2). Recent World Health Organization (WHO) evaluations on the short- and long-term benefits of breastfeeding came to the conclusion that there is solid evidence for various public health benefits for the infant, the mother, the household, and the community as a whole (3). The benefits for the infant include improve cognitive development, a decreased rate of necrotizing enterocolitis, common childhood infections, and a decreased risk of sudden death. Breastfeeding helps the mother return to her pre-pregnancy weight, improves birth spacing, and decreases the rate of chronic illness and breast cancer (3, 4). Moreover, optimally breastfed children contribute to improved human capital through a reduction in expenses on infant formula and increased opportunities for a more sustainable future (5).

The World Health Organization’s (WHO) recommendation for starting breastfeeding is within one hour after birth (6). Moreover, the World Health Organization and the United Nations Children’s Fund (UNICEF) recommends exclusive breastfeeding (EBF) for the first 6 months of life and continued breastfeeding until the child is 2 years of age (7). Early initiation of breastfeeding can reduce neonatal mortality by 33% (8). Different studies indicate that the late initiation of breastfeeding leads to high neonatal morbidity and mortality. According to a systematic review study, infants who initiated breastfeeding after one hour were 33% at risk of neonatal mortality (9). Moreover, studies in different parts of the world revealed that delayed breastfeeding increases the risk of mental and physical problems at different stages of life (8). Neonatal morbidity and mortality in infants who did not feed breast milk within one hour are increased by threefold when compared to infants who fed breast milk within one hour of birth (10–12).

Even though the evidence on breastfeeding suggests the existence of variation around the world, it is clear that the prevalence of early initiation of breastfeeding was higher in high-income countries than low- and middle-income countries (13). More recently, the United Nations renewed its commitment to improving child nutrition (including breastfeeding outcomes) worldwide through the United Nations Decade of Action on Nutrition (2016–2025) and the Sustainable Development Goals (SDG-2.2) of ending all forms of malnutrition (14, 15). Moreover, the Sustainable Development Goals (SDG) target reducing neonatal deaths to 12 per 1,000 live births and under-five deaths to less than 25 per 1,000 live births through eliminating preventable child deaths by the year 2030 (16).

In low- and middle-income countries, where access to clean water, adequate sanitation, and basic health and social services is often limited, the lower prevalence of early initiation of breastfeeding may increase various health problems (5, 7, 17). For example, a recent study from Nigeria indicated that an estimated 23% of diarrhea deaths among children under five were attributable to late breastfeeding in 2016 (18).

In Ethiopia, many subnational reports indicated that the prevalence of early initiation of breastfeeding was about 66.5%, which is below the Ethiopian Health Sector Transformation Plan (HSTP) target of 90% (19). The government of Ethiopia created the National Nutrition Program (NNP) I (2008–2015) and the NNP II (2016–2020) with a strong focus on multi-sectoral coordination of nutrition activities to expedite reductions in malnutrition (20). The importance of early initiation of breastfeeding was recognized together with other nutrition programs through national reforms and strategies on infant and young child feeding practices, especially after the launch of the health promotion program in 2003 (21). Accordingly, the prevalence increased from 52% in 2011 to 74.3% in 2016, but still fell below the plan of the Ethiopian Ministry of Health Sector Development Program, which targeted the 92% prevalence of early initiation of breastfeeding (22). A systematic review of mostly subnational studies reported that a mother’s occupation, education level, receiving guidance and counseling from health care workers, place of delivery, and mode of delivery were common factors associated with the early initiation of breastfeeding in Ethiopia (16). According to various studies, the WHO and the concerned United Nations organizations (UN) report that the factors associated with breastfeeding practice differ with socioeconomic, demographic, behavioral, and cultural factors of mothers, place and mode of delivery, professional counseling on breastfeeding, and obstetric and health service-related factors (23–25). Identifying factors associated with breastfeeding practices is essential to increasing the prevalence of early breastfeeding. Despite the fact that a number of single-level and descriptive studies have been done to analyze the trend, factors, and prevalence of early initiation time, exclusive breastfeeding, and duration of breastfeeding in Ethiopia, the prevalence is below the plan of the Ethiopian health sector, and more work is needed. Thus, in view of the above literature, this study targeted identifying individual and group-level factors associated with early initiation of breastfeeding using the data from the 2019 Ethiopia Mini Demographic and Health Survey (EMDHS).

## Materials and methods

### Data source and study design

This paper uses secondary data from the children’s data set of the 2019 Ethiopia Mini Demographic and Health Survey (EMDHS). The survey was population-based cross-sectional, and the data was downloaded from the Measure DHS Website^1^ after reasonable request, and data use permission was obtained from the institutional review board of the International Classification of Functioning (ICF). The survey conducted from March 21 to June 28, 2019, was the second EMDHS and the fifth DHS implemented in Ethiopia. The Ethiopian Public Health Institute (EPHI) carried out this survey in partnership with the Central Statistical Agency (CSA) and the Federal Ministry of Health (FMoH), with technical assistance from the ICF and financial and technical support from development partners.

### Sampling design

The sampling frame for the 2019 Ethiopia mini demographic and health survey was a complete list of the 149,093 enumeration areas (EAs), which was performed by the Central Statistical Agency (CSA). The average coverage for an enumeration area was 131 households. For the 2019 EMDHS, a two-stage stratified sampling design was used. Currently, Ethiopia is structured administratively into twelve nation-states and two city administrations. However, during the 2019 EMDHS exclusion, there were nine nation-states and two city administrations, which were then stratified into urban and rural areas except for Addis Ababa, which is entirely urban, to establish 21 sampling strata. To ensure the precision of sample selection, the allocation was done through an equal allocation where 25 EAs were selected from eight regions and each of the three main regions was assigned 35 EAs: Amhara, Oromia, and the Southern Nations, Nationalities, and Peoples’ Region (SNNPR). Among the 305 EAs selected in the primary stage, 93 were urban and 212 were rural, and in the second stage of selection, an equal probability systematic selection was used to select a fixed number of 30 families per cluster from the newly established household list. The survey interviewed 5,753 women of reproductive age (15–49) from a nationally representative sample of 8,663 households. In accordance with the denominator of early initiation of breastfeeding, the sample for this study was made up of last-born infants who were born within 24 months before the survey, which resulted in a total weighted sample of 2,125 infants.

### Variables in the study

In this study, we have considered two types of variables, namely, response and explanatory variables.

#### Response variable

The response variable in this study was early initiation of breastfeeding using the recommended definition of children born within 24 months before the survey who were put to the breast within one hour after birth (25). The response variable was dichotomized as early breastfeeding when the mother starts breastfeeding within one hour after birth and delayed breastfeeding when it exceeds one hour.


$$\mathrm{Breastfeeding initiation}=\{\begin{array}{c} 0,\mathrm{delayed initiation}\;\\ 1,\mathrm{early initiation} \end{array}.$$

#### Explanatory variables

The selection of explanatory variables was theoretically driven and supported by prior research with regard to factors influencing the early initiation of breastfeeding in Ethiopia. Therefore, the explanatory variables used in this study were the sex and gestational age of infants, which were potential infant-level predictors of early initiation of breastfeeding. Mother’s education level, age of mother, numbers of children ever born, preceding birth interval, mode of delivery, number of antenatal care visits, place of delivery, and wealth index were potential mother-level predictors of early initiation of breastfeeding, while residence was an enumeration-level predictor. The data was managed with SPSS 26 and analyzed using SAS 9.4. The data was weighted to take into account or adjust for disproportionate sampling and non-sampling responses and to increase the representativeness of the sample. Variable selection was made by the purposeful method, while a univariable analysis was made between a response variable and each predictor separately at a 25% level, then a multivariable analysis was performed at a 5% level between a response variable and all significant variables in the univariable analysis. The model selection was performed using deviance and log likelihood statistics due to the presence of nested models.

### The generalized linear model

The selection of any statistical analysis depends on the nature of the response and the explanatory variables. The standard linear regression analysis assumes a continuous response variable with a normally distributed random error term, but there are conditions where this assumption is clearly violated. For instance, when the response variable has a nominal and ordinal scale, there is no way to think about the normal distribution (26). Thus, the methods called generalized linear models were introduced by Nelder and Wedderburn in 1972 and revised in 1989 by McCullagh and Nelder to generalize linear models by the general mean structures and more general distributions (27).

### The multilevel binary logistic regression model

The binary logistic regression model is a generalized linear model where the outcome variables are measured on a binary scale. In most cases, those two categories of the response variable are noted as success and failure. If we define the binary response variable as


$$Y=\{\begin{array}{c} 1,\mathrm{if the outcome is success}\\ 0,\mathrm{if the outcome is failure} \end{array}$$

With the corresponding probabilities p(Y = 1) = π and p(Y = 0) = 1−π.

In this case, the response variable and the corresponding error term will assume a binomial distribution with a logit transformation rather than a normal distribution. If there are n such random variables $\;y_{1}$ , $\;y_{2}$ , $\;y_{3}$ , ………, $y_{n}$ with equal probability (π), then the random variable Y has the binomial (n, π) distribution as;


$$P\left(Y-y\right)=\left(\begin{array}{c} n\\ y \end{array}\right)\pi^{y}\;{\left(1-\pi \right)}^{n-y},y=0,1,2,3,\ldots ,n$$

Thus, binary logistic regression analysis expresses individual probabilities of success as a function of predictor variables as follows;


$$\pi_{i}=x_{i}^{\prime }\beta$$

Where the left hand side represents individual probability of success and the right hand side is the function of predictor variables. But the expressions on the left and right hand sides have different ranges; the left hand side has values between 0 and 1, while the right hand side assumes any real number. Thus, to overcome this problem, it is important to convert these probabilities into odds as follows;


$${\mathrm{odds}}_{i}=\frac{\pi_{i}}{1-\pi_{i}}$$

then we can take logarithms to obtain the logit link function, which has the effect of removing the floor restriction.


$$\mathrm{Logit}\;\left(\pi_{i}\right)=\log\;\left(\frac{\pi_{i}}{1-\pi_{i}}\right)=x_{i}^{\prime }\beta$$

Even though the above binary regression model provides us with the effect of several predictors on the response variable, due to the hierarchical nature of the data, where each infant is nested in a mother and each mother is nested in an enumeration area, the multilevel approach is recommended over the standard approach (28). If there are N predictors X at the child level, Q predictors Z at the mother level, and K predictors W at the enumeration area level, then the 3-level model for a binary outcome variable $\pi_{ijk}$ with a mean π is;


$$\begin{array}{l} \mathrm{Logit}\left(\pi \right)=\gamma_{000}+\gamma_{100}X_{\mathrm{ijk}}+\gamma_{01}Z_{\mathrm{jk}}+\gamma_{001}W_{k}+\gamma_{110}X_{\mathrm{ijk}}Z_{\mathrm{jk}}+\\ \gamma_{101}X_{\mathrm{ijk}}W_{k}+\gamma_{011}Z_{\mathrm{jk}}W_{k}+u_{1\mathrm{jk}}X_{\mathrm{ijk}}+u_{i1k}Z_{\mathrm{jk}}\;\;+\\ u_{0\mathrm{jk}}+u_{00k} \end{array}$$

Where, u_0jk_ and u_00k_ are the intercept variations at the mother and enumeration area level, respectively.

## Results

### Descriptive statistics

This study was conducted on the multilevel analysis of early initiation of breastfeeding among 2,125 infants who were born within 24 months before the 2019 Ethiopia mini demographic and health survey. Accordingly, the prevalence of early initiation of breastfeeding among the randomly sampled infants in the country was 72%. Among these randomly selected infants, 51.3% were male, and the remaining 48.7% were female. The average age of randomly selected mothers was 27.65 years, with minimum and maximum values of 15 and 49 years, respectively. Moreover, the average preceding birth interval, gestational age, and number of antenatal visits were 39.79 months, 33.6 weeks, and 3.08 visits, respectively. However, among these mothers, the majority (73.5%) belongs to rural residents, while the remaining 26.5% is urban residents. Most of the mothers (93.5%) used vaginal delivery. Regarding the place of delivery, the majority (54%) delivered their child in any of the health facilities. In addition, when compared to other categories of factors, the majority of these sampled mothers were illiterate unschooled (46.7%) and poor (41.0%). Moreover, the significance of the Chi Square value showed the existence of a significant association between the early initiation of breastfeeding and the corresponding predictors at a 5% level of significance (Table 1).

**Table 1:** Early initiation of breastfeeding and its socio-demographic and economic characteristics.

Variable	Categories	Frequency	Percent	$\chi^{2}$ -value(*p* value)
Breastfeeding	Early	1,530	72.0	
	Delayed	595	28.0	
Residence	Urban	562	26.5	89.2(<0.0001)
	Rural	1,562	73.5	
Mode of delivery	Caesarean	139	6.5	577.7(<0.0001)
	Vaginal	1986	93.5	
Education	No education	993	46.7	314.5(<0.0001)
	Primary	840	39.5	
	Secondary	190	8.9	
	Higher	102	4.8	
Place of delivery	Home	977	46	22.573(<0.0001)
	Health sector	1,147	54	
Wealth index	Poor	871	41.0	259.7(<0.0001)
	Middle	428	20.2	
	Rich	825	38.8	
Sex of child	Male	1,090	51.3	56.1(<0.0001)
	Female	1,035	48.7	

### Model selection

Model selection is the task of selecting the statistically best model that fits the statistical data among a series of candidate models. The intra-class correlation was computed from the intercept-only model for the purpose of identifying the proportion of variance explained by the grouping structure. Then the value demonstrated that the use of multilevel analysis, which takes into account the effect of such grouping structures, is more important than single-level analysis. Moreover, due to the hierarchical nature for the data, the presence of predictors at different levels in multilevel studies makes the model selection different from standard methods. Accordingly, different mixed-effects models were considered to analyse the early initiation of breastfeeding in Ethiopia. Due to the maximum value for log likelihood statistics and the minimum value for deviance, the random intercept model with fixed infant, mother, and enumeration area level predictors was the best fit to the data (Table 2).

**Table 2:** Multi-level model selection.

Fit statistics	Random intercept model				Random slop model	
	Null model	Level 1 predictors added	Level 2 predictors added	Level 3 predictors added	Level 2 predictors as random	Level 3 predictors as random
AIC	67.7	63.7	17.2	11.2	71.6	91.0
BIC	92.2	76.0	43.6	26.3	108.4	140.0
Log likelihood	−30.01	−29.9	−2.01	−1.6	−29.8	−37.5
Deviance	59.8	59.7	3.45	3.2	59.6	75.0

### The intercept-only model

The intercept-only model is the model fitted without any predictor variables. In multilevel analysis, the random effect results from the intercept-only model are important to indicate the necessity of such an analysis. The residual error variance at the mother and enumeration area levels was estimated to be 2.01472 and 4.8023, respectively, as a result of the maximum mean pseudo-likelihood estimation. We can conclude that there is more variation among the different enumeration areas (2.4219) than within the enumeration areas (0.4534). In a logistic regression model without over-dispersion, the lower level (child level) variance is not reported in the result, and it is automatically constrained to $\frac{\pi^{2}}{3}=3.29$ . The proportion of variance explained by the grouping structure could be discussed further when we calculate the intra-class correlation ( ρ ). In three-level models, the proportion of explained variance at the second and third levels can be identified by the intra-class correlation separately. Let the variances at the first, second, and third level are $\sigma^{2}\varepsilon_{0},$ $\sigma^{2}u_{0},$ and $\sigma^{2}v_{0}$ respectively (29).


$$\rho_{mother}=\frac{\sigma^{2}u_{0}}{\sigma^{2}\varepsilon_{0}+\sigma^{2}\;u_{0}+\sigma^{2}v_{0}}=\frac{2.01472}{3.29+2.01472+4.8023}=0.199$$

$$\rho_{EA}=\frac{\sigma^{2}v_{0}}{\sigma^{2}\varepsilon_{0}+\sigma^{2}\;u_{0}+\sigma^{2}v_{0}}=\frac{4.8023}{3.29+2.01472+4.8023}=0.475$$

This result implies that 19.9% of the variation in early initiation of breastfeeding was at the mother’s level, 47.5% was at the enumeration area level, and the remaining 32.6% was at the infant’s level (Table 3).

**Table 3:** Parameter estimation for the intercept only model.

Effect	$\hat{\beta }$	SE	*t* Value	*p* > |*t*|	Lower	Upper
Estimates for fixed effects						
Intercept	4.7226	0.2439	19.37	<0.0001	4.2444	5.2009

### Variable selection

When modeling with many independent variables, of which only a few are likely to be important, finding an appropriate subset of explanatory variables for the model is called variable selection.

### Univariable analysis

The first step in a purposeful variable selection procedure is a univariable analysis. This step concerned the analysis of each predictor with the early initiation of breastfeeding at a 25% level to “screen” out potentially significant variables for consideration in the multivariable model. Based on this analysis, all the predictors except sex of infants and wealth index were statistically significant at a 25% level and not considered in multivariable analysis (Table 4).

**Table 4:** Univariable analysis.

Variable	Num DF	Den DF	*F* value	*p* > *F*
Residence	1	2,123	71.0	<0.0001
Sex of child	1	2,123	0.23	0.6305
Wealth index	2	2,123	0.55	0.4435
Mode of delivery	1	2,123	17.90	0.0018
Place of delivery	1	2,133	15.12	0.031
Education	3	2,122	55.07	<0.0001
Preceding birth interval	1	2,124	14.82	0.0022
Number of children	1	2,124	31.02	<0.0001
Gestational age	1	2,124	75.09	<0.0001
Number of visits	1	2,124	78.92	<0.0001
Age	1	2,124	55.98	<0.0001

Num DF, Degrees of Freedom for the numerator of F distribution; Den DF, Degrees of Freedom for the denominator of F distribution.

### Multivariable analysis

This is the second step of the purposeful variable selection procedure, concerned with the analysis of all the significant predictors of early initiation of breastfeeding. Accordingly, results from the final multilevel binary logistic regression model revealed that age of mothers, place of residence, number of antenatal care visits, preceding birth interval, gestational age of infants, mother’s education level, mode of delivery, place of delivery, and the interaction of residence and mode of delivery were the significant predictors of early initiation of breastfeeding in Ethiopia. The random effect estimates revealed that there was more variation at higher levels (mothers and enumeration area) than at lower level (infants) (Table 5).

**Table 5:** Parameter estimates for multilevel binary logistic regression model.

Variable	Categories	$\hat{\beta }$ (Exp( $\hat{\beta }$ ))	SE	*p* > |*t*|	95% CI for exp. (β)	
					Lower	Upper
Estimates for fixed effects						
Intercept		4.9201	0.3619	<0.0001	4.2104	5.6298
Education	No education	0	–	–	–	–
	Primary	0.1311(1.14)	0.0566	0.0407	1.0204	1.2738
	Secondary	0.4328(1.54)	0.0250	<0.0001	1.4678	1.6190
	Higher	0.9651(2.62)	0.07697	<0.0001	2.2574	3.0526
Residence	Rural	−0.4581(0.63)	0.05450	<0.0001	0.5684	0.7038
	Urban	0	–	–	–	–
Mode of delivery	Vaginal	0.1017(1.11)	0.0024	<0.0001	1.1019	1.1123
	Caesarean	0	–	–	–	–
Place of delivery	Health facility	0.4690(1.60)	0.0467	<0.0001	1.4585	1.7515
	Home	–	–	–	–	–
Preceding birth interval	In months	0.1665(1.18)	0.01369	<0.0001	1.1076	1.5451
Age	In years	−0.8254(0.44)	0.05655	<0.0001	0.3921	0.4894
Number of children	Count	0.05961(1.06)	0.1068	0.5769	0.8609	1.3086
Gestational age	In weeks	0.3187(1.38)	0.0368	<0.0001	1.2796	1.4782
Number of visits	Count	0.2338(1.26)	0.0120	<0.0001	1.2340	1.2934
Residence*mode of delivery	Urban/vaginal	1.0417(2.83)	0.0880	<0.0001	2.3850	3.3675
	Rural/caesarean	−0.4915(0.61)	0.0311	<0.0001	0.5756	0.6501
	Rural/vaginal	0.5928(1.81)	0.0491	<0.0001	1.6430	1.9918
	Urban/caesarean	0	–	–	–	–

exp (β) = Adjusted odds Ratio (AOR).

## Discussion

This study was conducted based on secondary data obtained from randomly selected mothers across the country. The prevalence of early initiation of breastfeeding among infants within 24 months before the 2019 Ethiopia Mini Demographic and Health Survey was 72%. This proportion is below the plan of the Ethiopian Ministry of Health Sector Development Program, which targets the prevalence of early initiation of breastfeeding at 92% (22). Moreover, this prevalence is lower than the recently reported prevalence of early initiation of breastfeeding in Burundi (86.19%), Ruanda (80.57%), and Malawi (78.38%) (30). This variation might happen due to the differences in the demographic and socio-cultural characteristics of the countries, such as the mother’s educational attainment, age, place of residence, place of delivery, mode of delivery, number of antenatal care visits, preceding birth interval, and gestational age of infants.

The estimated adjusted odds ratio for the age of mothers was exp. (−0.8254) = 0.44, implying that a one-year increase in the age of mothers reduced the odds of early initiation of breastfeeding by 56% (95% CI: 0.3921, 0.4894), while all other variables remained constant. The result was consistent with refs. (30–32), where increasing the age of mothers was associated with a delayed initiation of breastfeeding.

The estimated adjusted odds ratio for the number of antenatal care visits was exp.(0.2338) = 1.26 (95% CI, 1.2340, 1.2934), implying that one more antenatal care visit was associated with a 26% increment in the odds of early initiation of breastfeeding among mothers in Ethiopia, while all other variables remained constant. The result was consistent with refs. (31, 33), where there is a direct relationship between the numbers of antenatal care visits and the prevalence of early initiation of breastfeeding.

The estimated adjusted odds ratio for the preceding birth interval was exp.(0.1665) = 1.18, implying that a one-month increase in the preceding birth interval increases the odds of early initiation of breastfeeding by 18% (95% CI, 1.1076, 1.5451), while all other variables remained constant. The result was consistent with refs. (31, 33), where an increasing preceding birth interval was associated with a higher prevalence of early initiation of breastfeeding.

The estimated adjusted odds ratio for the gestational age of a child was exp.(0.3187) = 1.38, implying that a one-week increase in gestational age increases the odds of early initiation of breastfeeding by 38% (95% CI, 1.2796, 1.4782), while all other variables remained constant. The result was consistent with refs. (34, 35), where increasing gestational age of an infant was associated with a higher prevalence of timely initiation of breastfeeding.

The estimated adjusted odds ratio of early initiation of breastfeeding among rural mothers compared to urban mothers was exp. (−0.4581) = 0.63, indicating that the odds of early initiation of breastfeeding among rural women were 37% (95% CI: 0.5684, 0.7038) lower than the odds of early initiation of breastfeeding among urban mothers, keeping all other variables constant. The result was consistent with refs. (33, 36–38), where rural residents were less likely to initiate timely breastfeeding than mothers in urban areas.

The estimated adjusted odds ratio of early initiation of breastfeeding among mothers with vaginal delivery as compared to those with caesarean section was exp. (0.1017) = 1.11, indicating that the odds of early initiation of breastfeeding among mothers with vaginal delivery were 11% (95% CI: 1.1019, 1.1123) higher than those with caesarean section, keeping all other variables constant. The result was consistent with refs. (6, 31, 34, 36–39), where giving birth by caesarean section is associated with a lower prevalence of early initiation of breastfeeding than vaginal delivery.

The estimated adjusted odds ratio of early initiation of breastfeeding among mothers who bear their child at a health facility as compared to those at home was exp. (0.4690) = 1.60, indicating that the odds of early initiation of breastfeeding among mothers who bear their child at a health facility were 60% (95% CI: 1.4585, 1.7515) higher than those at home, keeping all other variables constant. The result was consistent with refs. (31, 33, 36, 37), where mothers who delivered with the assistance of health professionals were associated with a higher prevalence of early initiation of breastfeeding. Moreover, where a dedicated psychologist and trained professionals in human lactation are present in hospitals to inform and support mothers immediately after childbirth, there will be a significant increment in the percentage of early onset and not abandonment of breastfeeding (40). The odds of early initiation of breastfeeding among mothers with primary, secondary, and higher education were 14, 54, and 162%, respectively, higher than the odds of early initiation of breastfeeding among mothers with no education, keeping all other variables constant. The result was consistent with refs. (31, 33, 38, 41), where educated mothers were more likely to initiate timely breastfeeding than illiterate mothers. In order to reach a higher overall percentage of breastfeeding, training should focus on women with lower levels of education. This subgroup is the one that could benefit the most from information from the staff in the hospital, taking into account the previous misinformation on the subject and reducing disadvantages coming from low education levels (42).

The odds of early initiation of breastfeeding among rural mothers with caesarean sections were 39% lower than the odds of early initiation of breastfeeding among urban mothers with caesarean sections, keeping all other variables constant. The odds of early initiation of breastfeeding among urban and rural mothers with vaginal delivery were 183 and 81%, respectively, higher than the odds of early initiation of breastfeeding among urban mothers with caesarean sections. The result was consistent with refs. (6, 38), where childbearing by caesarean section was significantly associated with delayed initiation of breastfeeding compared to vaginal delivery given urban or rural residents.

### Strengths and weaknesses of the study

This study demonstrates how data collected through demographic surveys can be used. The methodology used to identify factors influencing early breastfeeding is correct and the results are in line with known results. However, the data must be integrated with the other variables that justify a delayed start of breastfeeding, which can however be collected by *ad hoc* surveys on clinical records.

## Conclusion

This study aimed to identify factors associated with the early initiation of breastfeeding in Ethiopia using a framework of multilevel analysis of the 2019 Ethiopia Mini Demographic and Health Survey data. The prevalence of early initiation of breastfeeding was 72%, which is below the plan of the Ethiopian Ministry of Health Sector Development Program, and close monitoring of each mother during birth was important. As the result revealed, there was more variation due to the grouping structure than infant-level variation, so analysis of such data using a single-level approach may ignore such variation. Moreover, age of mothers, place of residence, number of antenatal care visits, preceding birth interval, gestational age of an infant, mother’s education level, mode of delivery, place of delivery, and the interaction of residence and mode of delivery were the significant predictors of early initiation of breastfeeding in Ethiopia. The federal and regional stakeholders should focus on infant feeding strategies, especially modifiable factors like place of delivery, mode of delivery, counselling during antenatal care visits, and socioeconomic characteristics of mothers. Moreover, sufficient supportive care, especially for rural mothers who undergo caesarean sections, is needed to improve the prevalence of early initiation of breastfeeding.

## Data availability statement

The original contributions presented in the study are included in the article/supplementary materials, further inquiries can be directed to the corresponding author.

## Ethics statement

The studies involving humans were approved by International Classification of Functioning (ICF) institutional review board (IRB# 187138). The studies were conducted in accordance with the local legislation and institutional requirements. Written informed consent for participation was not required from the participants or the participants' legal guardians/next of kin in accordance with the national legislation and institutional requirements.

## Author contributions

NH: Conceptualization, Data curation, Formal analysis, Funding acquisition, Investigation, Methodology, Project administration, Resources, Software, Supervision, Validation, Visualization, Writing – original draft, Writing – review & editing. TK: Data curation, Methodology, Writing – review & editing. GH: Methodology, Software, Writing – review & editing.

## Footnotes

^1^ http://www.measuredhs.com
